# The Molecular Interplay Between Oral Microbiome and Oral Cancer Pathogenesis

**DOI:** 10.3390/ijms262010212

**Published:** 2025-10-21

**Authors:** Roxana-Nicoleta Urzică, Brigitte Crețu, Ana Căruntu, Săndica Bucurica, Alexandru-Titus Farcasiu, Laurențiu Mihai Ciupescu, Cristian Scheau, Constantin Căruntu

**Affiliations:** 1 Department of Physiology, “Carol Davila” University of Medicine and Pharmacy, 050474 Bucharest, Romania; urzicaroxana@yahoo.com (R.-N.U.); cristian.scheau@umfcd.ro (C.S.); costin.caruntu@gmail.com (C.C.); 2 Department of Oral and Maxillofacial Surgery, “Carol Davila” Central Military Emergency Hospital, 010825 Bucharest, Romania; brighy15@gmail.com; 3 Faculty of Dental Medicine, “Titu Maiorescu” University, 031593 Bucharest, Romania; 4 Department of Gastroenterology, “Carol Davila” University of Medicine and Pharmacy, 050474 Bucharest, Romania; sandica.bucurica@umfcd.ro; 5 Department of Gastroenterology, University Emergency Central Military Hospital “Dr. Carol Davila”, 010825 Bucharest, Romania; 6 Department of Prosthodontics, Faculty of Dentistry, “Carol Davila” University of Medicine and Pharmacy, 37 Dionisie Lupu Street, District 2, 020021 Bucharest, Romania; alexandru.farcasiu@umfcd.ro; 7 Institute of Hygiene and Veterinary Public Health, 041293 Bucharest, Romania; ciupescu_laurentiu@yahoo.com; 8 Department of Radiology and Medical Imaging, “Foisor” Clinical Hospital of Orthopaedics, Traumatology and Osteoarticular TB, 021382 Bucharest, Romania; 9 Department of Dermatology, “Prof. N.C. Paulescu” National Institute of Diabetes, Nutrition and Metabolic Diseases, 011233 Bucharest, Romania

**Keywords:** oral cancer, microbiome, carcinogenesis, periodontitis

## Abstract

The oral microbiome plays a key role in oral cancer pathogenesis through mechanisms such as chronic inflammation, dysregulated proliferation, and increased tumor invasiveness. Dysbiosis, frequently present in premalignant and malignant lesions, may initiate or accelerate malignant transformation. Oral squamous cell carcinoma (OSCC), representing over 90% of oral cancers and affecting more than 350,000 people worldwide each year, is strongly linked to microbial shifts. Common periodontal pathogens such as *Fusobacterium nucleatum* and *Porphyromonas gingivalis* are often enriched in OSCC. These bacteria may promote tumorigenesis by activating NF-κB and STAT3 pathways, suppressing apoptosis, and modulating host immune responses. Additional potential mechanisms include the production of reactive oxygen species (ROS) and genotoxins, inhibition of tumor suppressors such as p53, disruption of cell-cycle regulation via cyclin-dependent kinase pathway, and upregulation of β-catenin and toll-like receptor signaling. These molecular alterations cause DNA damage, immune surveillance evasion, angiogenesis, promoting tumor progression. Microbiota-modulating therapies, such as *Lactobacillus* probiotics, may complement standard treatments by restoring balance, boosting immunity, and limiting tumor growth. Engineered bacteriotherapy, microbiome-targeted immunomodulators, and microbiota-based diagnostics expand therapeutic options in oral cancer and, combined with advances in precision medicine, may support more personalized treatments and improved outcomes.

## 1. Introduction

Oral cancer is an aggressive pathology, very challenging from the therapeutic perspective, and a topic of great interest to the scientific community. According to the latest Globocan report, the incidence of oral cancer is on a continuous rise. In the year 2022, there were 4.0 newly reported cases per 100,000, while the mortality rate reached 1.9 cases [1].

Oral cancer is a multifactorial disease. Both genetic and non-genetic factors play a role in its pathogenesis [2]. Mutations in the *p53* gene, encoding a transcription factor known for its ability to suppress tumors, were commonly detected in oral cancer patients [3]. These changes promote the conversion of healthy epithelial cells, which gain malignant features [4]. In oral squamous cell carcinoma (OSCC), Retinoblastoma protein (pRB) is frequently inactivated through loss of expression or functional mutations, consistent with its role as a tumor suppressor [5]. Another genetic alteration linked with oral cancer refers to Cyclin-Dependent Kinase inhibitors CDKIs and their regulatory partners [6]. These molecules coordinate the cell cycle progression and changes in their levels can promote uncontrolled cell division and proliferation, thus promoting malignancy [7]. Among non-genetic factors, tobacco, alcohol consumption, betel quid chewing, poor oral hygiene, ultraviolet radiation, and infections with carcinogenic human papillomaviruses (HPV) are well-known for their risk of developing oral cancer [8,9]. Previous studies have reported that smokers may have up to a seven-fold higher risk and drinkers up to six-fold higher risk of oral cancer [10]. Moreover, the combination of these two behaviors significantly increases the risk, their negative effects being mutually potentiating [11,12,13]. A study conducted by Nouri et al. demonstrates that dysbiosis of the oral microbiota—particularly influenced by smoking and alcohol—contributes to cancer risk through reduced SCFA (short-chain fatty acids) levels and FFAR2 expression [14]. These changes promote inflammation via the TNFAIP8 and IL-6/STAT3 pathways [14].

Epidemiological studies have shown that poor oral hygiene, periodontal disease and tooth loss were correlated with oral cancer, providing indications that the oral microbial population may be involved in oral carcinogenesis [15,16]. Similar connections were confirmed in gastric cancer, linking the carcinogenic process with preexisting *Helicobacter pylori* [17] or *Campylobacter pylori* infections [18,19]. In this context, the oral microbiome and its potential connections with oral cancer occurrence and progression have incited the interest of the scientific community [20]. The purpose of this review is to analyze and correlate the current knowledge on the molecular mechanisms that could link the oral microbiome and oral carcinogenesis. Additionally, we review the current knowledge on the therapeutic potential of oral microbiome in the management of oral cancer patients. This combined focus on molecular mechanisms, epidemiology, and innovative therapeutic opportunities distinguishes our review from earlier publications, offering a comprehensive and translational outlook for both researchers and clinicians.

Throughout the manuscript, the terms microbiota and microbiome will refer to the bacterial population found in the oral cavity, except when stated otherwise. Standardized taxonomy was used with italics for bacterial genera and species, and capitalized letters for the phyla. A structured literature search was performed in PubMed, Scopus, and Web of Science up to June 2025 using the terms oral cancer, oral squamous cell carcinoma, periodontitis, oral microbiome, and dysbiosis. Only peer-reviewed articles written in English, including clinical and experimental studies, were considered. Reference lists of relevant papers were also screened. Data were extracted and thematically synthesized to integrate epidemiological findings, molecular mechanisms, and therapeutic perspectives.

## 2. General Characteristics of the Oral Microbiome

Humans are hosts to multiple microbial communities, species that can contribute to both health and disease. The human body is estimated to host ~30–40 trillion microbial cells, roughly equal to the number of human cells, with a microbial gene repertoire vastly exceeding that of the human genome [13,21]. The human microbiome starts to develop gradually before and after birth, and it is continuously changing as a result of internal and external stimuli [22]. Healthy bacterial microbiota and its homeostasis with the human host are essential for normal body function [23]. The microbiota can interfere with multiple physiological processes. It can modulate immune responses, influence food intake, affect the appetite, take part in vitamin biosynthesis, protect from exogenous pathogens, and even produce antimicrobial substances, known as bacteriocins [24,25]. Between individuals, the bacterial microbiota varies according to age, sex, and diet, in health and disease, in addition to geographical variations [26,27]. The oral cavity, with its diverse microenvironments and accessibility, serves as a powerful model to study microbial interactions, ecological succession, and host-microbe dynamics. With the aid of sequencing and imaging technologies, the oral microbiome will continue to provide critical insights into biofilm development, ecology, and pathogenesis, ultimately informing new therapeutic strategies [28].

Bacteria make up the majority of the oral microbiota, with fungi and viruses being present in smaller amounts [29]. Comprising over 770 different bacterial species, the mouth cavity is home to one of the most prolific and diverse microbial communities in the human body, second only to the gastrointestinal tract population [30]. The Human Oral Microbiome Database (HOMD) lists the most prevalent phyla found in the oral cavity of an adult: Firmicutes, Bacteroidetes, Proteobacteria, Actinobacteria, and Fusobacteria [31,32]. The main genus in the oral cavity is *Streptococcus*, followed by *Haemophilus*, *Leptotrichia*, *Porphyromonas*, *Prevotella*, *Cutibacterium*, *Staphylococcus*, *Veillonella*, and *Treponema* [33,34]. The human oral cavity is a collection of ecosystems, such as the tonsils, saliva, hard and soft palate, tongue, buccal mucosa, gingival sulcus, and natural or artificial teeth surfaces [25,32]. The composition and activity of the microbiome can undergo significant and quick changes due to various factors, such as alterations in pH, gene mutations, or bacterial interactions [35]. While the microbiota composition in all sites has similarities, slight variations can be detected on a smaller scale [35]. In healthy individuals, researchers found different bacterial profiles on soft and hard surfaces in the oral cavity [36]. A particularity of the oral microbiome is the presence of saliva, which helps the microorganisms migrate from one site to another [25]. The oral cavity is continuously exposed to exogenous microorganisms while breathing, eating, and drinking. As a result, it can be quite difficult to determine which species are commensal and which are only transient [25]. Under certain conditions, the microbial community can be altered, and a state of dysbiosis develops. It has been suggested that alterations in the oral microbiota might affect the human’s general status [37]. Changes in the oral bacterial composition were detected in numerous systemic diseases such as allergies, type 1 and type 2 diabetes, inflammatory bowel disease, acute infections, asthma, Alzheimer’s disease, and different types of cancer [27,38,39,40], emphasizing the weight of oral microbiome on general health. Furthermore, there is a strong interplay between oral and intestinal microbiota. The oral-gut microbiome axis reflects a dynamic interaction in which oral microbial translocation to the intestine, especially during dysbiosis, has been increasingly implicated in the pathogenesis and aggravation of systemic diseases [41]. Thus, it is essential to clarify which is the profile of a healthy oral microbiome, in order to understand changes that lead to disease.

## 3. The Connection Between Oral Microbiome and Oral Cancer

From the above-mentioned data, a correlation between oral microbiota and oral cancer can be expected. Sami et al. discussed the relationship between the oral microbiome and OSCC and the “who came first” concept. They emphasized two possible hypotheses: either a single or a collection of bacteria may be directly responsible for the carcinogenic process, leading to alterations of the microbiome that favor an oncobiomic environment that harms healthy epithelial cells (“bacteria before tumor”), or the presence of bacteria in the OSCC tumor environment is opportunistic, but can influence the progression of the tumor (“bacteria after tumor”) [20]. To further investigate this association, it is essential to explore the molecular processes that might link the oral microbiota with the host immune system and the corresponding signaling pathways involved in oral carcinogenesis.

**Figure 1:**
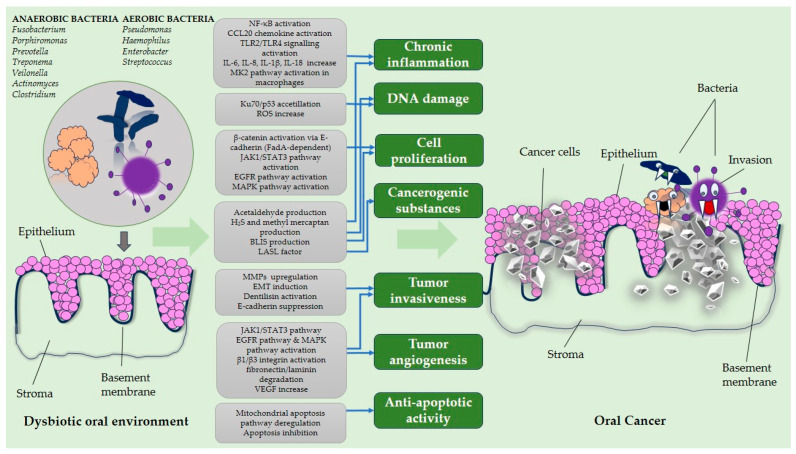
The molecular mechanisms linking the oral microbiome and oral cancer. Schematic representation of molecular mechanisms linking oral carcinogenic processes: inflammation, DNA damage, cell proliferation, angiogenesis, etc., with oral dysbiosis, and the progression from normal epithelial cells to malignancy. Original creation in PowerPoint by Urzica, NR (2025).

There are numerous mechanisms through which microorganisms can interact with components of the host’s immune system. The oral microbiome has been associated with carcinogenesis through the stimulation of chronic inflammation, cell proliferation, tumor invasiveness, and tumor angiogenesis, as well as through the inhibition of cellular apoptosis and the production of carcinogenic substances [8] (Figure 1).

Many species of anaerobic bacteria have been incriminated in oral carcinogenesis, the most studied being phylum Bacteroidotetes and Fusobacteriota, with the representative species *Porphyromonas* and *Fusobacterium* [20]. Alongside these, other species have also been detected in higher concentrations in oral cancer patients, such as *Prevotella*, *Peptostreptococcus*, *Veillonella*, *Haemophilus*, *Rothia*, and *Streptococcus* [42,43].

### 3.1. Oral Microbiome and Premalignant Lesions

Recent studies have shown that changes in the oral microbiome begin early in the process of malignant transformation. The healthy oral microbial population is predominantly bacterial, with bacteria accounting for over 90% of the community, while fungi represent approximately 0.1–1%, viruses 5–8%, and protists less than 0.1% of the total population [44]. Altered composition of the oral microbial populations has been detected even before the onset of oral cancer, in patients with premalignant lesions [45]. Most studies compared the affected mucosal surface against a contralateral non-affected site from the same subject. In oral leukoplakia (OLK) specimens, higher levels of members from the genera *Fusobacterium*, *Leptotrichia*, and *Campylobacter* were detected in comparison to the clinically normal contralateral tissue [46]. Several other studies reported similar findings in leukoplakia and erythroplakia lesions, with increased levels of *Leptotrichia*, *Campylobacter*, *Haemophilus*, *Rothia*, and *Fusobacteria*, and low levels of *Firmicutes* species [35]. Schmidt et al. identified an enrichment in the genera of *Fusobacteria* and *Bacteroides* in precancerous lesions [47]. These findings should be interpreted with caution, in the absence of healthy control comparison, and also potential biases related to oral site heterogeneity, or the field of cancerization phenomenon, common in oral carcinogenesis. The mechanisms underlying these changes are complex. In OSCC cell lines and tissue studies, *Fusobacterium nucleatum* has been associated with activation of E-cadherin/β-catenin signaling and NF-κB-mediated inflammatory responses [48], resembling mechanisms previously described in colorectal cancer [49]. *Leptotrichia* species may amplify TLR2/TLR4 signaling, contributing to the pro-inflammatory environment within the epithelia. Furthermore, in dysplastic epithelial cells, *Campylobacter* species, including *Campylobacter concisus* and *Campylobacter rectus*, can produce cytolethal distending toxin (CDT), inducing DNA alteration and cell-cycle arrest via ATM/Chk2 pathways [50,51]. *Haemophilus parainfluenzae* has been incriminated in promoting oxidative stress and upregulating matrix metalloproteinases (MMPs), which contribute to basement membrane degradation, promoting local expansion and invasiveness [52]. On the other hand, *Rothia* spp., through their production of acetaldehyde from ethanol metabolism, generate genotoxic effects that may accumulate in premalignant tissue [53].

Other studies assessing the bacterial population associated with oral premalignant lesions in comparison with healthy control subjects, reported similar findings. In OLK lesions, decreased levels of commensal bacteria were detected, and an enrichment of periodontal pathogens *Fusobacterium*, *Prevotella*, *Alloprevotella* and *Veillonella* [54]. Decsi et al. also found a rise in *Fusobacterium nucleatum* levels and a decrease in *Streptococcus mitis* in OLK patients [55], while Hashimoto et al. found that *Porphyromonas gingivalis* was predominant in both premalignant and malignant lesions [56]. Higher levels of *Porphyromonas* spp were also found in oral lichen planus (OLP), especially in severe erosive lesions [57]. *Fusobacteria* at the phylum level in conditions such as OLP and OLK points to their role in modulating cytokine expression and promoting epithelial–mesenchymal transition (EMT), setting the stage for a more aggressive behavior [47,58]. In a study including 25 benign lesions, 30 oral potentially malignant disorders (OPMD), and 35 OSCC specimens, using qPCR detection, *Prevotella melaninogenica*–positive OPMD showed significant upregulation of AKT2 expression (*p* = 0.042), a gene involved in cancer progression, whereas OSCC samples harbored *Streptococcus mitis* DUSP16 downregulation (*p* = 0.011), a gene linked to cellular signaling and proliferation [59].

It is challenging to interpret all these reports that vary in terms of study protocol, bacterial strains being assessed, or the number of patients. Despite all the drawbacks, it becomes visible that specific bacteria are constantly reported in association with oral premalignant lesions, namely Fusobacteria and Bacterioidetes strains. These findings suggest a potential role of these bacterial species in promoting malignant transformation through modulation of key oncogenic pathways and might be an argument favoring the hypothesis that the changes in the normal bacterial population could contribute to oral carcinogenesis.

### 3.2. Oral Bacterial Landscape and Oral Cancer

A wide range of bacterial strains have been investigated in oral cancer patients, with focus on the mechanism that could connect these two elements in terms of carcinogenesis and prognosis (Table 1). Many studies examining the biofilm present on the surfaces of OSCC tumors compared to adjacent healthy mucosa revealed higher quantities of anaerobic bacteria—*Veillonella*, *Fusobacterium*, *Prevotella*, *Porphyromonas*, *Actinomyces*, and *Clostridium*, together with several aerobic species *Haemophilus*, *Enterobacteriaceae*, and *Streptococcus β-haemolyticus* on tumor site [60,61]. It was postulated that the co-existence of specific pathogens—*Fusobacterium nucleatum* and *Porphyromonas gingivalis*—is associated with pro-tumorigenic mechanisms, including inflammation, immune evasion, and EMT [62]. However, aerobic bacteria represent an important component of the oral microbiota. Many of these microorganisms are commensal, but some species have been reported to act as opportunistic pathogens when there is a disbalance in the oral bacterial population [43]. Certain types of aerobic bacteria, such as *Streptococcus* spp., *Lactobacillus* spp., and *Pseudomonas aeruginosa*, have been linked to OSCC. Pushalkar et al. through genetic sequencing reported that *Streptococcus* sp. oral taxon *058*, *Peptostreptococcus stomatitis*, *Streptococcus salivarius*, *Streptococcus gordonii*, *Gemella hemolysis*, *Gemella morbillorum*, *Johnsonella ignava* and *Streptococcus parasanguinis* were common bacteria in the OSCC tumor [58].

In OSCC, *Fusobacterium nucleatum* promotes tumor development through the FadA adhesin, which modulates E-cadherin/β-catenin signaling, enhancing cell proliferation and invasion. It also triggers chronic inflammation via lipopolysaccharides (LPS) and flagella, fostering a tumor-promoting microenvironment [63]. *Pseudomonas aeruginosa* facilitates invasion and metastasis through LPS- and flagella-induced TLR/MyD88/ NF-κB activation, the ExoU cytotoxin stimulating IL-8/KC release, and the reduction in E-cadherin expression. Additionally, it can cause DNA double-strand breaks, leading to genomic instability [63]. It has the ability to convert nitrites into nitric oxide using its enzyme—c1 nitrite reductase [64]. Vyhnalova et al. investigated the potential role of *Pseudomonas aeruginosa* quorum-sensing molecules in oral carcinogenesis. Extrapolating from other tumors, experiments on in vitro cancer cell models identified factors such as LasI (AHL synthase) and LasR (transcriptional regulator) that might be involved in impairing cell adhesion and promoting an invasive or metastatic behavior [29]. In addition to the frequently studied genera *Fusobacterium* and *Porphyromonas*, recent studies have highlighted other taxa that may also play relevant roles in OSCC. In particular, *Capnocytophaga gingivalis* has been consistently reported at higher abundance in OSCC patients and has been suggested to promote tumor progression through EMT, indicating its potential as a microbial marker for early detection [65]. An observational cross-sectional study, based on 16S rRNA gene sequencing of saliva samples from healthy subjects, patients with OLK, oral submucous fibrosis, and OSCC, demonstrated distinct microbiome shifts [66]. *Neisseria* was identified as a core component of the oral microbiome across all groups but demonstrated a marked and statistically significant increase in abundance in OSCC compared to healthy controls, where it represented the most dominant genus (15.05%). This enrichment was positively correlated with higher frequency of risk habits such as tobacco and betel quid use. Importantly, *Neisseria* spp. are known to generate acetaldehyde, thereby providing a plausible mechanistic link between microbial dysbiosis, lifestyle-associated exposures, and oral carcinogenesis [66].

**Table 1:** Molecular mechanisms linking oral pathogenic bacteria to oral cancer.

Bacterium (Phylum)	Gram	Mechanism of Action	References
*Fusobacterium nucleatum* (Fusobacteriota)	+	FadA adhesin activates E-cadherin/β-catenin signaling; chronic inflammation via LPS and flagella; CCL20 activation; promotes proliferation, invasion, metastasis; DNA damage via Ku70/p53; activates TLR4 → p38 MAPK & NF-κB (p65); increases IL-6 and IL-8; Fap2 binds TIGIT, inhibiting NK cells.	[20,40,63,67,68,69]
*Porphyromonas gingivalis* (Bacteroidetes)	−	Inhibits apoptosis via JAK1/STAT3; increases invasion, migration, EMT; oxidative stress & DNA damage; chemoresistance; gingipains degrade ECM and inactivate complement; activates β-catenin/TCF; modulates miRNAs (e.g., miR-21); induces IL-6 and IL-8.	[29,68,70,71,72]
*Treponema denticola* (Spirochaetota)	−	Dentilisin enhances invasiveness; disrupts junctions; increases MMPs; activates β1/β3 integrins; degrades fibronectin/laminin; activates p38 MAPK & ERK1/2.	[72]
*Aggregatibacter actinomycetemcomitans* (Pseudomonadota)	−	Induces pro-inflammatory cytokines; produces H_2_S and methyl mercaptan; promotes proliferation/migration/angiogenesis; MK2 pathway; LtxA toxin induces apoptosis; stimulates IL-1β via NLRP3; activates osteoclastogenesis.	[73,74,75]
*Prevotella intermedia* (Bacteroidetes)	−	Dysbiosis/inflammation; extracellular proteases degrade immunoglobulins & complement; activates IL-8 and TNF-α via TLR2/TLR4.	[76,77]
*Peptostreptococcus stomatis* (Bacillota)	+	Promotes inflammation; enriched in OSCC tumor tissue; possible synergy with other anaerobes.	[58]
*Parvimonas micra* (Bacillota)	+	Correlated with advanced stage & lymphatic spread; promotes inflammation; stimulates IL-6; activates NF-κB; biofilm cooperation with *F. nucleatum*.	[68]
*Streptococcus anginosus* (Bacillota)	+	Produces acetaldehyde; induces apoptosis; DNA damage via aldehydes; activates IL-8 and TNF-α; upregulates COX-2/PGE2; increases VEGF-mediated angiogenesis.	[78]
*Pseudomonas aeruginosa* (Pseudomonadota)	−	LPS/flagella → TLR/MyD88/NF-κB; ExoU induces IL-8/KC; LasI disrupts E-cadherin; DNA double-strand breaks; nitrite reductase; exotoxin A inhibits protein synthesis; ROS generation; elastase degrades ECM.	[29,63,64]
*Haemophilus influenzae* (Pseudomonadota)	−	Higher abundance in advanced OSCC; activates TLR2/TLR4 → IL-8; induces NETs, sustaining inflammation.	[68]
*Fusobacterium periodonticum* (Fusobacteriota)	−	Linked to advanced stage & lymphatic spread; stimulates IL-1β and IL-18 via inflammasome.	[68]
*Streptococcus constellatus* (Bacillota)	+	Enriched in advanced stages; involved in inflammation; lactate production supports anaerobes.	[68]
*Filifactor alocis* (Bacillota)	+	Increased in advanced OSCC; oxidative stress resistance via superoxide dismutase; evades phagocytosis; sustains inflammation via IL-1β.	[68]
*Veillonella* spp. (Bacillota—class Negativicutes)	−	Associated with OSCC; lactate metabolism to propionate/acetate; alters pH; stimulates IL-6/IL-8.	[68]

Yang et al. studied the oral microbiome in relation to OSCC progression across TNM stages. They reported significantly higher abundances of *Fusobacterium periodonticum*, *Parvimonas micra*, *Streptococcus constellatus*, *H. influenzae*, and *Filifactor alocis* in advanced stages, as supported by LEfSe analysis (LDA score > 3.0). Notably, *F. periodonticum* and *P. micra* were positively associated with lymphatic spread. Consistent with observations from premalignant lesions, most of the genera enriched in tumor tissues are species typically associated with periodontal disorders [68].

### 3.3. Periodontitis Bacteria and Oral Cancer

It is widely accepted that poor oral hygiene and periodontitis have been associated with an increased risk for oral cancer [69,79]. A meta-analysis by Zeng et al. suggested that periodontitis may be associated with a higher likelihood of OSCC, reporting a pooled odds ratio of 2.63 (95% CI: 1.68–4.14) across case–control and cohort studies, most of which adjusted for smoking and alcohol consumption [80]. Complementing these epidemiological data, Katz et al. demonstrated the presence of *Porphyromonas gingivalis* within gingival squamous cell carcinoma specimens, supporting the concept of direct bacterial colonization of tumor tissues, although without quantifying cancer risk [70]. The similarities in terms of bacterial characteristics between oral cancer and periodontal disease additionally support this correlation [81,82]. Periodontal bacteria stimulate the production of pro-inflammatory mediators, promoting chronic inflammation, cell proliferation, and tumor angiogenesis. The study of Zhao et al. reported significant alterations in the bacterial composition and gene functions in OSCC samples, especially for species associated with periodontitis, such as *Fusobacterium*, *Dialister*, *Peptostreptococcus*, *Filifactor*, *Peptococcus*, *Catonella*, and *Parvimonas*, which exhibited a substantial increase in OSCC samples [82]. Chronic bacterial inflammation leads to hypermethylation and silencing of CDKN2A (p16INK4a), often mediated by the upregulation of DNMT1 and DNMT3B, enzymes, stimulated in inflammatory microenvironments [83]. Microbial components can also activate EGFR signaling pathways, fostering epithelial hyperproliferation and angiogenesis [84].

Pathogens such as *Fusobacterium nucleatum* and *Porphyromonas gingivalis* have been associated with oxidative stress and DNA damage in oral epithelial cells, processes that may contribute to TP53 alterations and compromise thetumor-suppressive functions of p53 [85]. These pathogens can affect specific intracellular pathways, promote cell survival, activate oncogenic pathways, increase cell migration and invasion, and facilitate metastasis [48,52]. Investigations conducted on a murine model have shown that chronic bacterial co-infection with these two pathogens can promote malignant transformation via direct interaction with oral epithelial cells through Toll-like receptors [48]. Moreover, *Porphyromonas gingivalis* increases tumor invasiveness and resistance to chemotherapy [67]. It can also inhibit apoptosis through overstimulation of the JAK1/STAT3 signaling pathway, participating in the regulation of mitochondrial apoptosis, cellular differentiation, migration, and proliferation [29,71]. Katz et al. investigated the existence of *Porphyromonas gingivalis* in tissue samples obtained from patients with gingival squamous cell carcinoma. When compared to normal gingival tissue, the tumor samples revealed greater levels of *Porphyromonas gingivalis*, exceeding 33% on immunohistochemical staining [70]. In light of these observations, the authors postulated a possible connection between *Porphyromonas gingivalis* and the development of malignant lesions. Similarly, *Fusobacterium nucleatum* was shown to have a major impact on aggressive tumor behavior through the activation of chemokines, such as chemokine (C-C motif) ligand 20 (CCL20) [52,64].

Several studies explored the role of *Aggregatibacter actinomycetemcomitans*, a bacterium associated with aggressive periodontitis, in oral cancer pathogenesis. *Aggregatibacter actinomycetemcomitans* is recognized to generate the production of pro-inflammatory cytokines, hydrogen sulfide, and methyl mercaptan, which can lead to inflammation, stimulate cell proliferation, cell migration, and tumor angiogenesis [20,74]. On a murine model, using Mk2+/+ and Mk2−/− mice, Herbert et al. analyzed the activity of this bacterium and reported that through the path of MAPK-activated protein kinase 2 in macrophages it contributes to the regulation of chemokine signaling and, as a result, promotes inflammation and subsequent bone loss [73]. Furthermore, the combination of chemotherapy and radiotherapy, along with the inhibition of MK2, has demonstrated potential in managing the growth and dissemination in different types of tumors [75]. Another periodontal pathogen that may be involved in the process of oral carcinogenesis is *Treponema denticola,* an oral anaerobic spirochete, that is known to support the overexpression of dentisilin, a chymotrypsin-like protease that is correlated with tumor invasiveness [72].

### 3.4. The Link Between Other Oral Microbial Components and Oral Cancer Development

Although bacteria dominate the oral microbial population (>90%), fungi, mainly *Candida albicans* (~0.1–1%), can contribute to molecular events underlying oral carcinogenesis [72]. Under homeostatic conditions, cross-kingdom interactions stabilize the oral niche, but in dysbiosis they acquire pathogenic properties. *Candida albicans* produces acetaldehyde from ethanol, a genotoxic metabolite that induces DNA adducts, chromosomal breaks, and microsatellite instability [52]. This genotoxic effect is amplified by bacteria such as *Streptococcus* and *Rothia*, which also catalyze acetaldehyde formation [53]. In parallel, co-colonization of *Candida* with *Fusobacterium nucleatum* or *Porphyromonas gingivalis* enhances NF-κB and STAT3 signaling, increases IL-6 and TNFα expression, and promotes epithelial proliferation with reduced apoptosis [58]. Bacteria also influence fungal morphology and persistence. *Streptococcus gordonii* and *F. nucleatum* upregulate *Candida* adhesins and hyphal transition, facilitating a robust biofilm architecture. Hyphal networks, in turn, shield bacteria from immune recognition and promote persistence of inflammatory signaling. This cross-protection enhances ROS release, activates MMPs, and triggers EMT, via β-catenin and TGF-β signaling [86]. Overall, the interaction between bacteria and fungi in the oral microenvironment appears to potentiate inflammation, DNA damage, and epithelial dysregulation, suggesting that future therapeutic approaches should target not only bacterial dysbiosis, but also inter-domain microbial interactions to effectively reduce oral cancer risk.

Viruses also play a critical role in the development of oral cancer. Among them, high-risk human papillomaviruses (HPVs), especially HPV-16 and HPV-18, are most strongly associated with OSCC. Their oncoproteins E6 and E7 promote malignant transformation by binding to p53 and RB1, thereby impairing DNA repair, inhibiting apoptosis, and releasing E2F transcription factors, which drive uncontrolled cell-cycle progression [87]. Furthermore, HPV infection has been shown to activate the PI3K/AKT/mTOR and MAPK pathways, contributing to enhanced cell proliferation, angiogenesis, and resistance to therapy [88]. Epstein–Barr virus (EBV) has also been implicated in oral carcinogenesis through latent membrane protein 1 (LMP1), which stimulates NF-κB and JAK/STAT signaling, promoting inflammation, EMT, and immune evasion [89]. EBV infection may also upregulate pro-angiogenic factors such as VEGF, further enhancing tumor progression [90]. Additionally, interactions between viruses and bacteria may create a synergistic oncogenic microenvironment. For example, HPV-positive OSCC lesions co-infected with dysbiotic bacteria, including *Fusobacterium nucleatum*, demonstrate increased pro-inflammatory cytokine expression, oxidative stress, and accelerated EMT, thereby amplifying tumor aggressiveness [91]. Taken together, the molecular insight emphasize that viruses, particularly HPV and EBV, act not only as direct oncogenic drivers but also as modulators for host–microbe interactions. Their role complements bacterial and fungal dysbiosis, underlining the need for integrative approaches when studying the multifactorial pathogenesis of OSCC.

To conclude this section, we can state that even though many aspects remain unclear in regard to oral carcinogenesis and the influence of the surrounding environment with the associated dysbiosis, all the above mentioned studies revealing alterations in the oral microbial landscape, detectable as early as from the precancerous status, confirm that the oral microbial population in general, and bacterial in particular, play a significant role in the pathogenesis of oral cancer [86]. Considering the heterogeneity of the currently available studies in terms of design, sample types, preclinical and clinical data (Table 2), a critical appraisal is essential when comparing these results. Lifestyle confounders like smoking, alcohol, betel quid chewing, and poor oral hygiene can simultaneously drive both dysbiosis and carcinogenesis, and this can further complicate data interpretation. The development of OSCC is ultimately determined by the host cell, immunological response, and metagenomic, and transcriptomic, events that occur in the oral microbiome and impact the normal function of the oral epithelial cells. Although no evidence supports a primary initiating role of the microbiota, it remains a plausible but unproven contributor to malignant transformation and cancer progression, requiring longitudinal validation.

### 3.5. Oral Microbiome and Gastrointestinal Cancers

The mechanisms through which the oral microbiome influences neoplastic processes are highly complex, acting both locally and systemically. Its impact is not limited to the oral cavity, with numerous studies highlighting a connection with the development and progression of various cancers. The oral cavity, as the initial segment of the digestive tract, harbors a large microbial community whose imbalance impacts the whole gastrointestinal system. Thus, the concept of oral microbiota implications in gastrointestinal cancers has evolved (Table 3). Alongside the local and systemic alterations, some species of oral microbiota, such as *Neisseria* genus, *Candida glabrata*, or *Streptococcus*, favor malignancy, by an increased production of carcinogenic acetaldehyde from alcohol enzymatic conversion [77,92,93,94]. This effect is furthermore enhanced when smoking is associated [68]. Esophageal cancer was linked to an imbalanced oral microbiome, mostly in patients with lower species diversity in saliva [64], revealing different patterns of oral dysbiosis. The behavior of certain species, such as *Streptococcus anginosus,* in cell cultures was examined, showing a contribution to cellular apoptosis [78]. Despite significant differences in oral microbiome constituents in esophageal cancer, including specific strains with clear carcinogenic features, the causal relationship still requires validation in future research [77].

For gastric cancer, some studies have reported a more abundant tongue film, with unspecific features. Other studies found a higher proportion of nitrate-reducing species such as *Haemophilus parainfluenzae* and *Nitrospirae*, against the species that reduce N-nitroso compounds, incriminating the N-nitroso metabolites pathway in gastric carcinogenesis [76,94,95]. Huang et al. reported increased values for bacterial DNA of Cyanobacteria species and *Streptococcus* genera in the saliva of patients with gastric cancer [95].

**Table 3:** Oral dysbiosis and other aerodigestive cancers.

Organ	Bacterial Type and Changes	Molecular Mechanisms	Reference
Esophageal cancer	↑ *Prevotella melaninogenica*, *Streptococcus mitis*, *Porphyromonas gingivalis*, *Capnocytophaga gingivalis*, *Firmicutes*, *Negativicutes*, *Selenomonadales*, *Prevotellaceae*, *Prevotella*, *Veillonellaceae* *Bulleidia*, *Catonella*, *Moryella*, *Corynebacterium*, *Peptococus*, *Cardiobacterium*	Acetaldehyde production and ROS production Apoptosis induction and modulates tumor microenviroment Promotes DNA damage EMT Chemoresistance	[76,96,97,98,99]
Gastric cancer	↑ *Haemophilus parainfluenzae*, *Nitrospirae* Cyanobacteria species, *Streptococcus* ↓ *Anaerovorax*, *Bulleidia*, *Peptostreptococcs*	N-nitroso compounds Acetaldehyde production	[94,95]
Pancreatic cancer	↑ *Neisseria elongata*, *Streptococcus mitis*, *Porphyromonas gingivalis*	Cytokine production DNA damage Acetaldehyde production ROS production	[76,77,100,101]
Colorectal cancer	↑ *Fusobacterium nucleatum*, *Prevotella intermedia* ↓ *Haemophilus*, *Micromonas Prevotella Heterobacterium*, *Neisseria Streptococcus*	Inflammation Dentilisin → tumor invasiveness Mycrobiome dysregulation DNA damage	[76,77]

↑ increased expression; ↓ decreased expression.

The species associated with colorectal cancer (CRC), such as *Prevotella intermedia* and *Fusobacterium nucleatum,* were found in samples from gingival plaque, saliva, or oral swabs [76]. In addition, increased species of *Peptostreptococcus*, *Parvimonas*, *Gemella* genera, and *Treponema denticola* were also described in CRC [77]. A special consideration was given to pro-inflammatory and carcinogenetic species of *Fusobacterium nucleatum*, which were found 400-fold higher in tumoral tissue, and were associated with chemoresistance in CRC [77,102].

The deficient oral health and hygiene were associated with pancreatic cancer, and notable differences in salivary microbiota were reported [77,103]. While *Fusobacterium* was found equivocally linked to pancreatic cancer, the *Neisseria elongata*, *Streptococcus mitis*, *Prophyromonas gingivalis*, and *Aggregatibacter actinomycetemcomitans* species were predominant in the saliva of these patients [77,100,101].

We can observe that bacterial strains detected in higher concentrations in oral cancer patients were also linked to other types of aerodigestive malignancy. Thus, samples collected from saliva or oral cavity swabs could serve as accessible tools for analyzing and characterizing the oral microbiome changes, and future studies might confirm and validate their role as non-invasive biomarkers for the timely detection of gastrointestinal cancers.

## 4. Therapeutic Strategies Involving Oral Microbiota in the Management of Oral Cancer

In terms of therapy, surgery, radiotherapy, chemotherapy, and more recently immunotherapy, constitute the main approaches in the fight against oral cancer [104]. Brachytherapy using low radiation doses can also be implemented as a viable and more conservative treatment option for specific cases [105].

Gendicine, a recombinant human p53 adenovirus, was approved in China by the State Food and Drug Administration (SFDA) in 2003 for the treatment of head and neck squamous cell carcinoma, including oral cancer, usually in combination with radiotherapy. It remains the only p53-based gene therapy with regulatory approval, based on supporting evidence from local clinical studies [106]. But the quest for novel, effective therapeutic alternatives is an ongoing process. Natural substances such as vitamin A [107], luteolin, resveratrol, kaempferol, quercetin, piperine [108] or cannabinoids are being assessed for their role in oral cancer management [109,110,111]. However, despite all efforts, the prognosis has not improved dramatically in recent decades [112]. The overall recurrence rate in oral cancer was estimated at 30% [113,114]. Patients with oral cancers often experience functional and esthetic impairment, with a major negative impact on the quality of life compared to other cancers [115]. These individuals face body image issues [116], and are at risk for severe malnutrition [117], and depressive disorders [118]. Oral cancer patients have the second-highest rates of suicide among oral cancer survivors, following pancreatic cancer patients [119]. These prognostic reports highlight the need for more effective preventive, diagnostic and therapeutic strategies in oral cancer [120,121,122].

### 4.1. Bacterial Potential as an Antitumor Agent

It was first documented around 150 years ago that tumor lesions might be treated with microorganisms [123]. In light of the current global malignant growth, developing new therapeutic approaches for this illness is imperative. Thanks to genome sequencing and genetic engineering, tumor-specific bacterial strains have been found, reviving interest in the use of bacteria as anti-cancer strategies. This concept has been vigorously explored using several *Clostridium* species [124], anaerobic, Gram-positive rods that can produce spores [125]. It was noted that tumors from gas gangrene patients would recede after contracting a clostridial infection [126]. Thus, sterile filtrates from *Clostridium histolyticum* were tested to treat advanced cancers [127]. Parker and colleagues infected tumor-bearing mice with clostridial spores and reported tumor lysis [128]. Salmonella strains, motile, Gram-negative bacteria that grow well in both oxygenated and hypoxic tumor areas [129], have also been tested for tumor-selective therapy [130]. Genetically stable chromosomal deletion of the purl and msbB genes was used to attenuate *Salmonella typhimurium* pathogenicity. The new strain, called VNP20009, is under investigation for its anti-tumor potential, with encouraging results [131,132]. Using attenuated *Salmonella choleraesius* in several malignancies showed delayed tumor growth and improved survival. Lee et al. administered *Salmonella choleraesuis* carrying an endostatin expression vector in an experimental mouse model with melanoma or bladder cancer and reported a 40–70% reduction in tumor growth and prolonged overall survival [133]. Immunohistochemical analysis revealed decreased intratumoral microvessel density, downregulated VEGF expression, and enhanced infiltration of CD8^+^ T cells [133]. The nonmotile Gram-positive bacterium *Streptococcus* has also been investigated as an anti-tumor agent, after the physicians W. Busch and F. Fehleisen reported regression of tumors after accidental infections by erysipelas [134]. After inducing a *Streptococcus pyogenus* infection in a patient with an inoperable sarcoma, it was reported that within a week, the primary tumor and the cervical lymph nodes had shrunk in size [135].

### 4.2. Probiotics as Part of the Oral Cancer Management Strategy

The impact of the microbiome on cancer therapy has been studied over the last years, suggesting the potential beneficial effects, including an enhanced effectiveness of anti-cancer treatments in different malignancies [136,137,138]. In oral cancer, the research is restricted to preclinical studies and should be interpreted with caution. In mouse models with tongue cancer, the addition of *Lactobacillus fermentum* increased the levels of G-CSF, GM-CSF, IgG, IgM, IL-4, IL-12, TNF-alpha, and IFN-gamma. Also, it improved the phagocytic ability and the local tissue antioxidants, and decreased the expression of malondialdehyde, a damaging product [139]. *Lactobacillus plantarum* induced apoptosis in an experimental model on KB oral cancer cell cultures through the upregulation of PTEN and MAPK signaling pathways, and was proposed as a probiotic adjuvant in OSCC treatment [20,140]. Several in vivo animal model studies and in vitro studies evaluated the effects of probiotics against oral cancer, defining inhibitory effects on oral carcinogenesis in probiotics, such as *Lactobacillus plantarum* and *Lactobacillus salivarius Ren* [141]. Another study conducted by Shen et al. suggests that *Neisseria sicca*, in combination with *Corynebacterium matruchotii*, exerts a protective role against OSCC by promoting genome stability, activating ATR–CHK1 signaling and NLRP3/GSDMD-mediated pathways, and suppressing pro-inflammatory responses, thereby highlighting its potential as a probiotic in OSCC management [142]. Furthermore, an interesting perspective is represented by capsaicin, the main ingredient of hot peppers, which demonstrated an antitumor effect on oral cancer cells and a significant antimicrobial action [143,144,145].

**Figure 2:**
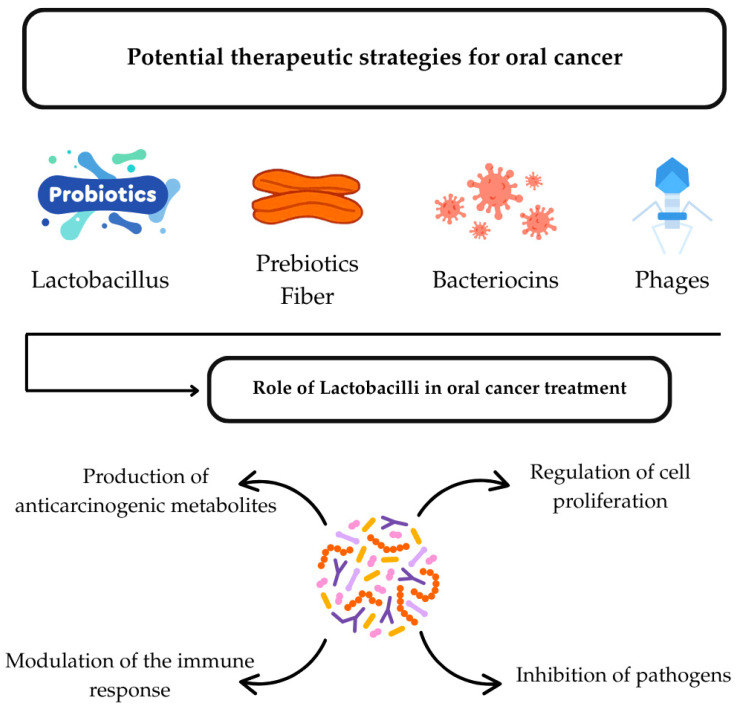
Potential therapeutic strategies associated with microbiota in oral cancer. Schematic representation of the four elements investigated for their potential therapeutic role in oral cancer, including the possible mechanisms of action. Original created in Canva.com software (https://canva.com), Urzica, NR (last accessed on 15 August 2025).

It is now possible to employ targeted methods to control or eradicate major oral pathogens by using probiotic strains that are specifically adapted for the oral cavity [146] (Figure 2). Probiotics can inhibit the growth of specific microbial pathogens, helping to restore the healthy oral microbiota [147]. In addition, some strains produce compounds, known as bacteriocins and bacteriocin-like inhibitory substances (BLIS) that can prevent the growth of competing bacteria [148]. BLIS K12 and BLIS M18, the originators of oral probiotics that produce BLIS, belong to the species *Streptococcus salivarius* [148]. It is becoming more evident that the future of using oral probiotics will likely go beyond addressing the immediate effects of oral microbiome imbalance and will target various systemic disorders that affect the human host [148]. The beneficial outcomes of probiotics are associated with their capacity to modulate the immune system, protecting the host against inflammation and apoptosis induced by different pathogens [149], interfering with their molecular interactions with other commensal or pathogenic microbes [150], and their influence on microbial or host cell by-products [151]. Antibiotic use in inflammatory disorders like gingivitis and periodontitis was considered, but given the adverse effects, including disruption of the oral and intestinal flora, the risk-benefit balance was not favorable. Furthermore, it was shown that prolonged antibiotic regimens for periodontal disease in patients with a subsequent diagnosis of cancer correlated with a shortened lifespan [152].

Without any doubt, there is a big interest in probiotics as an adjuvant to chemotherapy [148]. Moreover, there is mounting evidence that the derivatives of probiotics, such as prebiotics, synbiotics, and postbiotics have a significant influence on carcinogenesis by regulating the oral microbiome [153]. Probiotics have been shown to produce a biofilm that possesses antibacterial activity against oral pathogens, whereas their derivatives support growth and increase the benefits of probiotics [153]. Furthermore, studies have indicated that postbiotics may enhance the effectiveness of chemotherapy, while also reducing its associated side effects [154]. SCFA, microbial cell fraction, functional proteins, metabolites, cell lysates, and polysaccharides are components of postbiotics [155]. Polysaccharides exhibited the most prominent anti-cancer activity among all the compounds [156]. In addition, it was proved that the degradation of the basement membrane is the first step of metastasis [157]. As a result, the suppression of the VEGF-MM2/9 signaling pathway can reduce the metastatic abilities of cancer cells [158]. A study conducted on 99 OSCC patients, before surgical resection of the tumor and other adjuvant therapy, showed that their salivary microbiome profile had a higher abundance of potentially pathogenic bacteria compared to healthy controls and speculated on the idea of using prevention measures, such as pre- or probiotics, salivary substitutes, or dietary counseling [159]. Moreover, microbiota transplant is becoming a popular process to restore or generate a healthy microbiome [160]. While fecal microbiota transplantation has shown potential in malignancies associated with gut microbial dysbiosis [161], there is currently no clinical evidence assessing the effects on oral microbiota or supporting its efficacy in OSCC. Furthermore, the risks—including transfer of antibiotic-resistant bacteria and opportunistic pathogens—remain a major safety concern. Recent studies suggest that postbiotics may represent a significantly safer alternative for restoring microbial balance [162]. These approaches should be regarded as investigational, and their relevance for OSCC management requires further validation (Table 4).

## 5. Conclusions

The human oral microbiome, comprising a complex ecosystem of bacteria, fungi, and viruses, plays a fundamental role in maintaining oral and systemic health. Recent research has increasingly revealed its critical involvement in the pathogenesis of malignancies, including oral cancer, not merely as a bystander but as a potential active driver of disease onset, progression. This review has highlighted how specific shifts in microbial communities, particularly an increase in anaerobic bacteria *Fusobacterium nucleatum* and *Porphyromonas gingivalis*, can trigger chronic inflammation, promote tumor invasion, suppress apoptosis, and ultimately contribute to oral carcinogenesis. Beyond oral cancer, the oral microbiota has shown systemic relevance, being linked to other gastrointestinal malignancies. These associations suggest that oral dysbiosis may serve not only as a risk factor, but potentially as an early biomarker for cancer detection.

Furthermore, research has shown that a controlled manipulation of the microbiota might have potential as an adjuvant treatment strategy in malignancy. This article underscores emerging therapeutic approaches that leverage the microbiome, from genetically engineered bacterial vectors to probiotics and postbiotics. These interventions offer exciting prospects as adjuncts to conventional cancer therapies, improving immunomodulation, drug responsiveness, and overall patient outcomes.

Despite these promising insights, significant questions remain. The causal relationship between dysbiosis and carcinogenesis is unclear, and translating microbiome research into clinical practice demands rigorous validation. Future studies should prioritize longitudinal cohort designs, standardized microbial profiling methods, and controlled clinical trials to clarify causal links and identify reliable microbial biomarkers. In conclusion, while a deeper understanding of the oral microbiome’s role in carcinogenesis holds promise for improving early detection and guiding innovative therapeutic approaches, current evidence remains preliminary and requires rigorous validation.

## Figures and Tables

**Table 2:** Oral Cancer Microbiome Detection Methods.

Detection Method	Description	Common Applications	Sample Type	Microbiome Target	Detectable Microorganisms	References
**16S rRNA Gene Sequencing**	Targets bacterial ribosomal RNA genes to identify and quantify microbial taxa	Profiling bacterial communities in OSCC	Saliva, tissue biopsy	Bacterial taxonomy (genus/species level)	*Fusobacterium*, *Streptococcus*, *Prevotella*	[47,66,68]
**Metagenomics**	Whole-genome sequencing of microbial DNA from samples	Comprehensive microbial diversity analysis	Saliva, tissue biopsy	Bacterial genes, pathways, and species composition	*Porphyromonas*, *Actinomyces*; *Candida albicans*; HPV, EBV	[43,64,87]
**Transcriptomics**	Analyzes RNA transcripts to study gene expression patterns	Identifying active microbial genes in tumors	Tissue biopsy	Bacterial gene expression profiles	*Streptococcus mitis*; *Fusobacterium nucleatum* *Candida* spp.	[59]
**Proteomics**	Studies protein expression and interactions	Detecting microbial proteins linked to cancer	Tissue biopsy, saliva	Bacterial protein markers and functional pathways	*Treponema denticola*; *Candida albicans*	[52,72]
**Metabolomics**	Measures metabolites produced by microbes	Linking microbial metabolism to tumorigenesis	Saliva, blood, tissue	Bacterial metabolic products and signatures	*Prevotella*, *Lactobacillus*; *Candida*	[14,53]
**Quantitative PCR (qPCR)**	Quantifies specific microbial DNA/RNA sequences	Validation of microbial biomarkers	Saliva, tissue biopsy	Targeted bacterial genes or species	*Fusobacterium*, *Streptococcus mitis*; *Prevotella melaninogenica* HPV	[59,68]
**Salivary Biomarker Analysis**	Detects microbial and host-derived markers in saliva	Non-invasive early detection of OSCC	Saliva	Bacterial enzymes, DNA fragments, or metabolites	*Capnocytophaga*, *Neisseria*; *Candida*; HPV	[36,52,66,87]

**Table 4:** The therapeutic potential of oral microbiota in oral cancer.

Species	Gram	Mechanism	Effects	References
*Lactobacillus* spp.	+	Inhibits the binding of other bacteria to the epithelial cells	Arrests bacterial growth	[163]
*L. fermentum*	+	Anti-inflammatory properties Phagocytosis	↗G-CSF ↗GM-CSF ↗IgG ↗IgM ↗IL-4 ↗IL-12 ↗TNF-alpha ↗IFN-gamma ↘Malondialdehyde ↘Damaging products	[139,164]
*L. plantarum*	+	Apoptosis in OSCC	↗PTEN ↗MAPK	[114,115]
*L. salivarius Ren*	+	Cancer cell inhibition	N/A	[116]
*Neisseria sicca* *Corynebacterium matruchotii*	−/+	Pyroptosis Anti-inflammatory properties Tumor growth inhibition	↘Cyclin D1 ↘NF-κB ↘IL-6 ↘CD4+ T cells ↘M2 macrophages	[142]

↗ increases secretion; ↘ decreases secretion.

## Data Availability

No new data were created or analyzed in this study. Data sharing is not applicable to this article.

## Abbreviations

The following abbreviations are used in this manuscript:

CRC	Colorectal Cancer
EMT	Epithelial–Mesenchymal Transition
HOMD	The Human Oral Microbiome Database
LasL	Las autoinducer synthase
LasR	Las Regulator
MMP	Metalloproteinase
OLK	Oral Leukoplakia
OPMD	Oral Potential Malignant Disorder
OLP	Oral Lichen Planus
OSCC	Oral Squamous Cell Carcinoma
SCFA	Short-Chain Fatty Acids
